# Real-World Verification of Artificial Intelligence Algorithm-Assisted Auscultation of Breath Sounds in Children

**DOI:** 10.3389/fped.2021.627337

**Published:** 2021-03-23

**Authors:** Jing Zhang, Han-Song Wang, Hong-Yuan Zhou, Bin Dong, Lei Zhang, Fen Zhang, Shi-Jian Liu, Yu-Fen Wu, Shu-Hua Yuan, Ming-Yu Tang, Wen-Fang Dong, Jie Lin, Ming Chen, Xing Tong, Lie-Bin Zhao, Yong Yin

**Affiliations:** ^1^Department of Respiratory Medicine, Shanghai Children's Medical Center, Shanghai Jiao Tong University School of Medicine, Shanghai, China; ^2^Paediatric AI Clinical Application and Research Center, Shanghai Children's Medical Center, Shanghai, China; ^3^Child Health Advocacy Institute, China Hospital Development Institute of Shanghai Jiao Tong University, Shanghai, China; ^4^Tuoxiao Intelligent Technology Company, Shanghai, China

**Keywords:** auscultation, breath sound, electronic stethoscope, artificial intelligence, children

## Abstract

**Objective:** Lung auscultation plays an important role in the diagnosis of pulmonary diseases in children. The objective of this study was to evaluate the use of an artificial intelligence (AI) algorithm for the detection of breath sounds in a real clinical environment among children with pulmonary diseases.

**Method:** The auscultations of breath sounds were collected in the respiratory department of Shanghai Children's Medical Center (SCMC) by using an electronic stethoscope. The discrimination results for all chest locations with respect to a gold standard (GS) established by 2 experienced pediatric pulmonologists from SCMC and 6 general pediatricians were recorded. The accuracy, sensitivity, specificity, precision, and F1-score of the AI algorithm and general pediatricians with respect to the GS were evaluated. Meanwhile, the performance of the AI algorithm for different patient ages and recording locations was evaluated.

**Result:** A total of 112 hospitalized children with pulmonary diseases were recruited for the study from May to December 2019. A total of 672 breath sounds were collected, and 627 (93.3%) breath sounds, including 159 crackles (23.1%), 264 wheeze (38.4%), and 264 normal breath sounds (38.4%), were fully analyzed by the AI algorithm. The accuracy of the detection of adventitious breath sounds by the AI algorithm and general pediatricians with respect to the GS were 77.7% and 59.9% (*p* < 0.001), respectively. The sensitivity, specificity, and F1-score in the detection of crackles and wheeze from the AI algorithm were higher than those from the general pediatricians (crackles 81.1 vs. 47.8%, 94.1 vs. 77.1%, and 80.9 vs. 42.74%, respectively; wheeze 86.4 vs. 82.2%, 83.0 vs. 72.1%, and 80.9 vs. 72.5%, respectively; *p* < 0.001). Performance varied according to the age of the patient, with patients younger than 12 months yielding the highest accuracy (81.3%, *p* < 0.001) among the age groups.

**Conclusion:** In a real clinical environment, children's breath sounds were collected and transmitted remotely by an electronic stethoscope; these breath sounds could be recognized by both pediatricians and an AI algorithm. The ability of the AI algorithm to analyze adventitious breath sounds was better than that of the general pediatricians.

## Introduction

Although non-invasive methods for the diagnosis and follow-up of lung diseases have undergone rapid development, the auscultation of breath sounds with a stethoscope remains a key part of the initial examination of lung diseases. The stethoscope has the advantages of being non-invasive, easy to use, affordable, and non-radioactive, and stethoscope-based examinations can be repeated quickly, making the device especially suitable for use for pediatric patients. It is well-known that the results of traditional auscultation are subjective and depend on the clinical experience and auditory perception ability of the physician; additional limitations of traditional auscultation include the inability to save or share the sound signal, its poor repeatability, and the inability to continuously monitor the breath sounds, among others.

To compensate for the above shortcomings of the traditional stethoscope, we used an electronic stethoscope to collect the breath sounds of children with pulmonary diseases in a real respiratory ward environment and used an AI algorithm to automatically identify the collected breath sounds. The breath sounds were distinguished into crackles, wheeze and normal sounds. Our study found that the results of the AI algorithm were substantially consistent with those of experienced pediatric pulmonologists.

China has a vast territory and a large population; however, the distribution of health resources throughout the country is not evenly distributed and differing pediatricians have variable clinical abilities. The combination of an electronic stethoscope and an AI algorithm may aid in conducting telemedicine sessions, improve the lung auscultation skills of general pediatricians, and become an important tool for child health management and chronic disease follow-up for families in the future.

Although non-invasive methods such as chest X-ray, chest computed tomography (CT) scan, and chest ultrasound have developed rapidly in the diagnosis and follow-up of pulmonary diseases, the auscultation of breath sounds with a stethoscope is still a key part of any initial examination. Following the invention of the stethoscope by Laennec in 1861, auscultation has become an important part of the diagnostic process, and the stethoscope has gradually evolved into the most commonly used instrument in the medical and healthcare industry.

The stethoscope has the advantages of being non-invasive, easy to use, affordable, and non-radioactive, and stethoscope examinations can be repeated quickly, making this tool especially suitable for children with respiratory symptoms. Among its benefits, lung auscultation can improve the sensitivity of the diagnosis of pneumonia in children (1), help in building a discrimination model of admission signs for drowning children (2), and be applied to recognizing wheezing and judging the presence of an asthma attack (3), and changes in breathing sound parameters can indirectly reflect the clinical scenario, such as limited airflow in the lung (4). However, traditional auscultation technology has obvious limitations in clinical application, including the dependence of the auscultation results on the clinical experience and auditory perception ability of physicians, which is strongly subjective; the inability to save and share the auscultated sound signal; poor repeatability; and the inability to continuously monitor breath sounds. Especially in terms of the subjective aspect of the auscultation results, a previous study confirmed that the accuracy of lung auscultation of physicians with different levels of experience or different specialties was significantly different; the accuracy of the respiratory specialists was the highest, while that of family doctors and medical students was generally lower (5), potentially reducing the value of auscultation in making clinical diagnoses.

Faced with the above situation, scientists invented the electronic stethoscope and have attempted to apply it to the real clinical environment. To some extent, the electronic stethoscope overcomes some of the shortcomings of the traditional stethoscope in sound data storage and sharing, but it does not improve the accuracy and efficiency of breath sound recognition (6). In recent years, artificial intelligence (AI) algorithms have been applied to the processing and recognition of breath sounds, among which the most commonly used algorithms include artificial neural networks, Gaussian mixture models and support vector machines, and some promising achievements have been reported (7).

The purpose of this study is to quantitatively evaluate the recognizability of adventitious breath sounds according to an AI algorithm in a real pediatric clinical environment.

## Materials and Methods

### Study Population

The study was carried out from May to December 2019 at the pediatric respiratory department in Shanghai Children's Medical Center (SCMC). This study was based in a hospital environment, and the auscultation recordings were collected in a real inpatient department. The inclusion criteria were as follows: (1) pediatric inpatients of either sex who were between 28 days and 18 years of age; (2) patients whose auscultations were described as normal, crackles or wheeze by a pediatric pulmonologist with at least 10 years of work experience in SCMC; (3) patients whose parent or guardian provided consent; and (4) patients who could cooperate with the process and keep quiet during the auscultation recording collection. Auscultation results described as both crackles and wheeze by the pediatric pulmonologist were excluded. For each patient, auscultation recordings were collected by an electronic stethoscope from different points on the chest. In addition to the breath sound recordings, we also recorded clinical data, including patient demographic characteristics and diagnosis.

### Auscultation Recording Procedure

**Figure 1:**
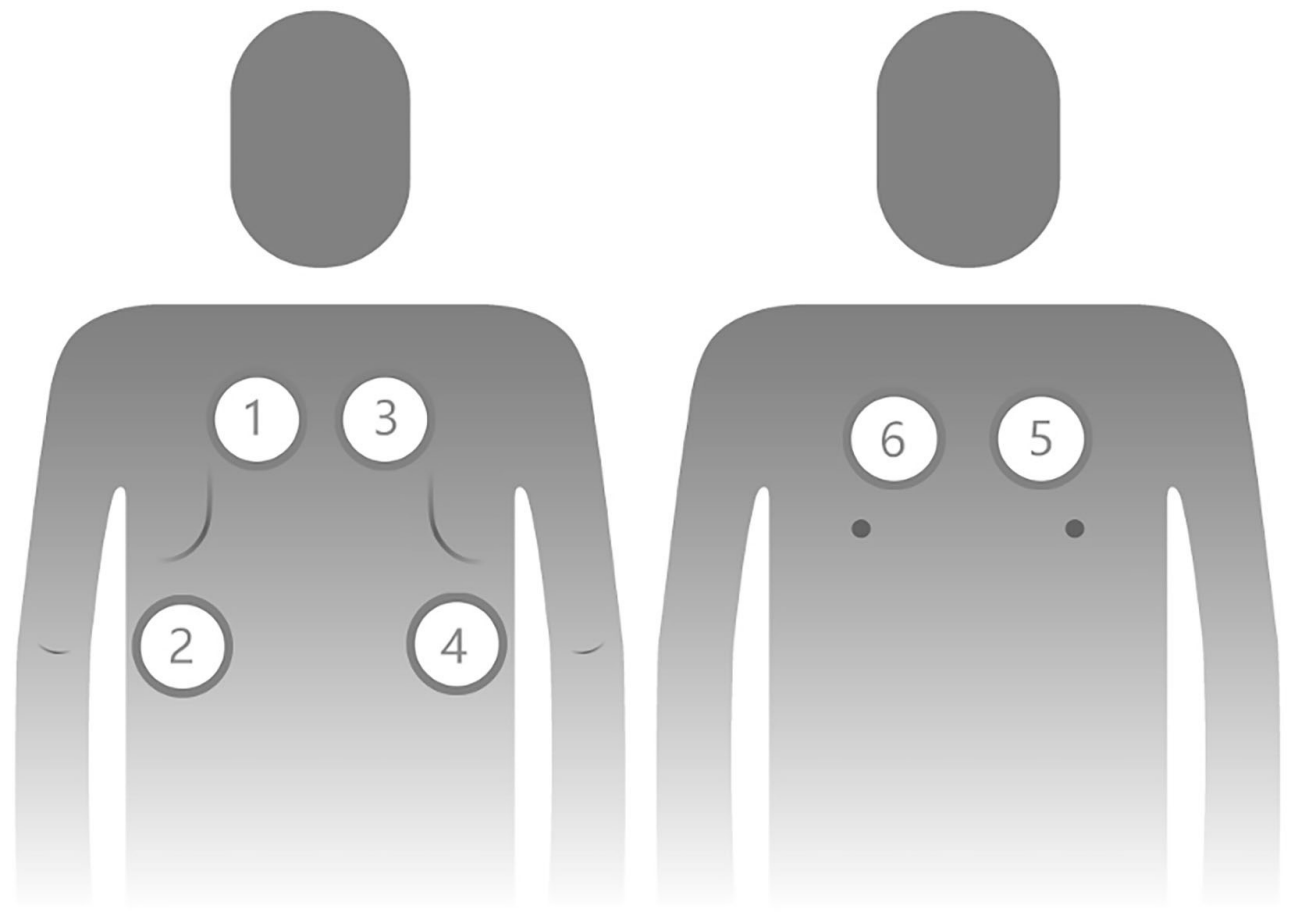
Order and localization of lung auscultation points for pediatric patients. Points 1 to 4 are on the posterior side of the chest, and points 5 and 6 are on the anterior side of the chest.

**Figure 2:**
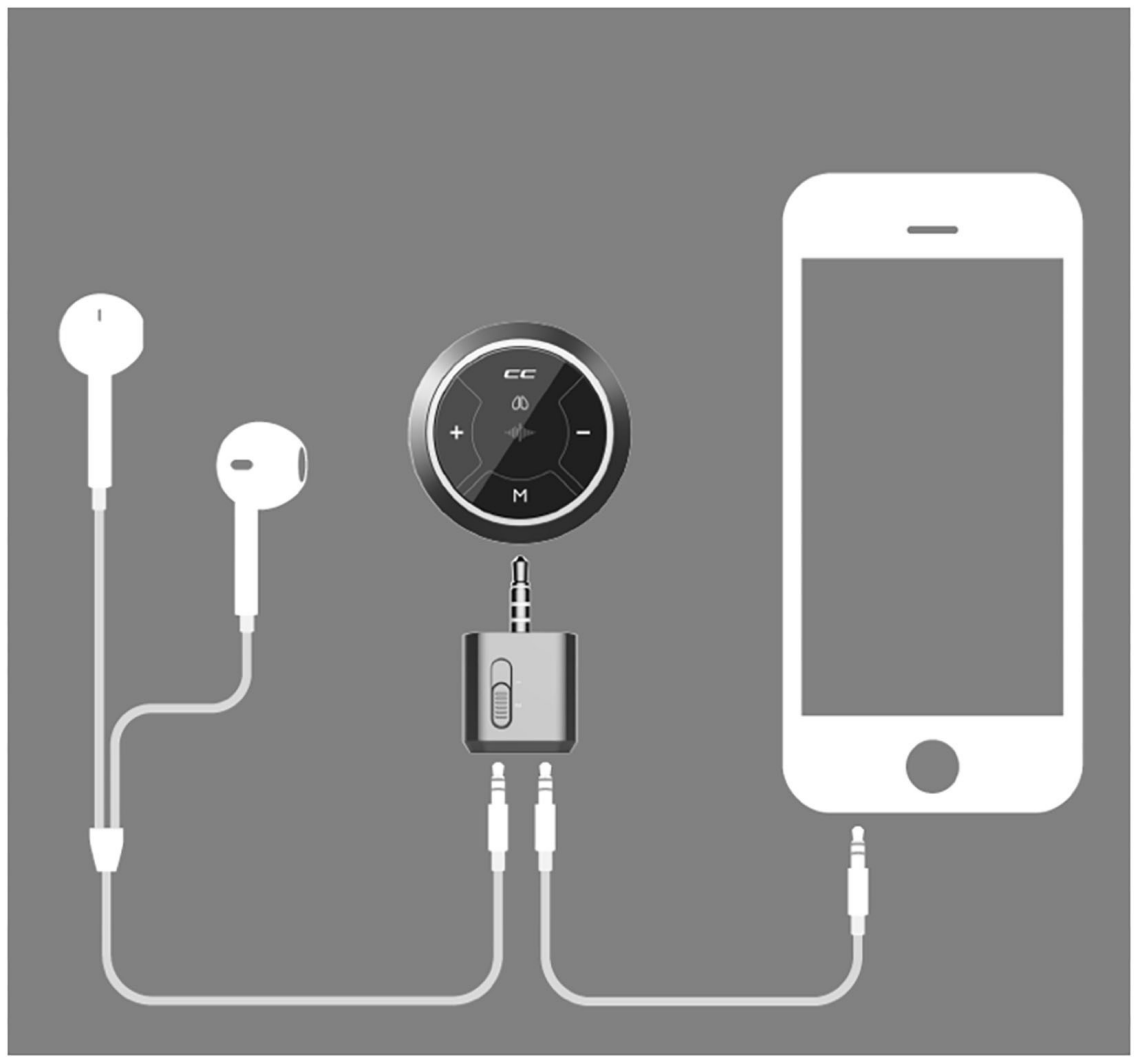
The electronic stethoscope connects to the smartphone and uploads the lung auscultation recordings to the cloud.

We trained three respiratory specialist nurses from SCMC to collect breath sounds with a Class II CE-marked electronic stethoscope (Yunting model II, Tuoxiao, Shanghai, China) prior to the study. The training included the recording and uploading processes and supervised practice. We collected a set of 6 recordings that included each child's chest (two) and back (four) and covered all parts of the lung (Figure 1). Auscultation was recorded for 9 s to obtain at least two breathing cycles per location (8). The electronic stethoscope and a smartphone were connected by a data wire, and the recordings were uploaded to the cloud through a smartphone app (Figure 2). Children's breath sounds were collected in a relatively quiet environment in the ward while the child was in either a sitting or supine/prone position. During the collection process, the children and their parents were asked to remain quiet; the children did not need to breathe deeply.

### Description of the Auscultation Recordings and Gold Standard Establishment

We asked experienced pediatric pulmonologists from SCMC, general pediatricians from various communities and the AI algorithm to describe the recordings as crackles, wheeze or normal breath sounds. Before the study began, all the participants were trained and assessed according to the nomenclature advised by the Europe Respiratory Society (9). At the same time, we provided both the age and sex of the patients with every recording to the participants for analysis. We recruited six general pediatricians from various communities with more than 5 years of work experience to mark all of the auscultation recordings independently.

**Figure 3:**
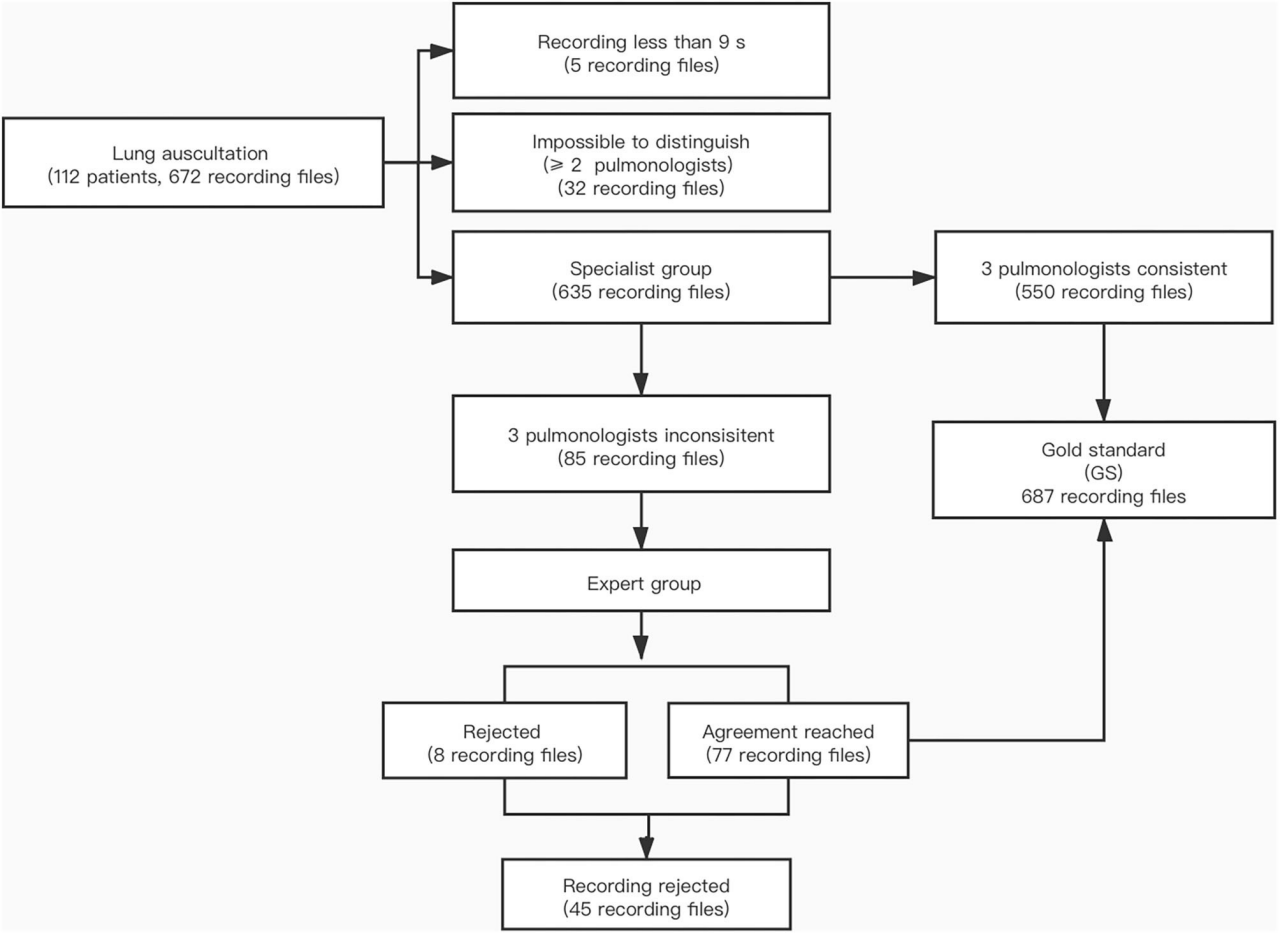
Flow chart of the establishment of the gold standard. Expert group: 2 pediatric pulmonologists with more than 20 years of work experience. Specialist group: 6 pediatric pulmonologists with at least 10 years of work experience.

The classification of adventitious sounds is subjective, and the results depend on the clinical experience of the physician. Therefore, it was necessary to establish a gold standard (GS) of breath sounds for this study. We selected eight pediatric pulmonologists with at least 10 years of work experience at SCMC to take part in establishing the GS. Among them, two pulmonologists with more than 20 years of experience composed the expert group, and the other six pulmonologists constituted the specialist group. Meanwhile, we randomly separated the collected auscultation recordings into two parts, each of which was marked by any three pulmonologists in the specialist group; in other words, each breath sound was marked three times independently by different pulmonologists. If the opinions of the three pulmonologists in the specialist group were consistent, the recordings were directly qualified as the GS. When two or more pulmonologists in the specialist group were unable to distinguish the recording or if the results of all three pulmonologists were inconsistent, the specialists directly rejected using the recordings as the GS. If two of the three pulmonologists' opinions in the specialist group were inconsistent, the expert group would discuss the findings for further verification and decide if the recording meets the requirement for the GS. The steps of the establishment process of the GS are shown in Figure 3. The study protocol was approved by the Institutional Review Board of SCMC (approval No. SCMCIRB-K2019056-1).

### Artificial Intelligence Algorithm

**Figure 4:**
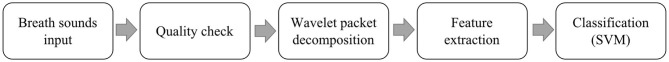
Major components of the automated adventitious breath sound detection performed by the AI algorithm.

The breath sound detection algorithm was developed by Tuoxiao Intelligent Technology Company, Shanghai, China, without the use of the recordings in the current study for training. It is designed with a record-upload-analyse mode and uses a cloud server. First, analysis of the characteristics of clinical crackle audio data revealed that the crackle was a pertinent discontinuous signal, with a duration of <20 ms and a peak magnitude more than two times the average magnitude. Analysis of the wheeze revealed that its average duration was usually more than 500 ms, and the peak portion of the ringing sound fragment over a 160 ms window was greater than the average of the filtered signal after performing low-pass filtering with a 200 ms Hamming window (10). The obtained clinical breath sound recordings were pre-processed according to the above features, and then the features were extracted using wavelet packet decomposition (11, 12). Finally, a support vector machine (SVM) was trained, and the parameters were obtained to establish an AI algorithm model. The algorithm comprises several major components in this study (Figure 4). The SVM had been trained and validated on a set of 6,234 and 6,423 real recordings, respectively. The performance of the AI algorithm showed an accuracy, sensitivity and specificity of 90.3, 88.3, and 92.3% in the detection of crackles and 87.1%, 86.7% and 87.5% in the detection of wheeze, respectively.

### Statistical Analysis

A confusion matrix was used to measure the performance of the AI algorithm, in which the values of accuracy, recall, precision, specificity, and F1-score were included as important evaluation parameters. Accuracy is the ratio of the correct samples predicted to the total number of samples and was used to represent the predictive ability of all the classification models. Recall is the proportion of correctly recognized true positives, also known as sensitivity. Precision quantifies the proportion of true positive-class predictions made from all positive predicted samples in the database. Specificity is the proportion of correctly recognized true negatives. The F1-score is the harmonic mean of recall and precision.

One-way ANOVA (chi-square test) was used to calculate whether there was a significant difference between the AI algorithm and each general pediatrician. The agreement levels between the AI algorithm and GS and between the AI algorithm and the individual general pediatricians were compared by the weighted kappa (κ) test and the corresponding 95% confidence intervals (CIs). Agreement among the general pediatricians across all the breath sound recordings was evaluated using Kendall's coefficient of concordance (Kendall's W). The parameters including accuracy, recall, precision, specificity, and F1-score of the breath sound recordings discrimination were calculated for the AI algorithm and the general pediatricians for the different locations on the chest and for different age groups.

## Results

### Demographics

A total of 112 patients were recruited for this study. The median age at the time of visit was 12.5 months (P_25_-P_75_, 5 to 41.8 months), and 82 patients were male (73.2%). The patients' ages were distributed as follows: ≤ 12 months, 56 (50%); between 13 and 60 months, 43 (38.4%) and > 60 months, 13 (11.6%). The patients' recordings were classified into crackles (159, 25.4%), wheeze (204, 32.5%) and normal breath sounds (264, 42.1%). The primary diagnoses were pneumonia (67%), bronchitis (13.4%), bronchiolitis (13.4%) and asthma attack (4.5%) (Table 1).

**Table 1:** Participant demographics.

**Characteristics**	**Study group (*n* = 112)**
Sex, *n* (%)	
Male	82 (73.2)
Female	30 (26.8)
Age group, months^*^	12.5 (5, 41.8)
<12, *n* (%)	56 (50.0)
12–60, *n* (%)	43 (38.4)
> 60, *n* (%)	13 (11.6)
Weight, kg^*^	9.6 (7.0, 15.8)
Height, cm^*^	75.0 (63.3, 100.0)
Primary diagnosis, *n* (%)	
Pneumonia	75 (67.0)
Bronchitis	15 (13.4)
Bronchiolitis	15 (13.4)
Asthma attack	5 (4.5)
Foreign body aspiration	1 (0.9)
Bronchiolitis obliterans	1 (0.9)

* *Median and quartiles [median (25%,75%)]*.

### Agreement

A total of 672 auscultation recordings were collected, and 627 (93.3%) were analyzed by the AI algorithm. Recordings were rejected if the duration of the recording was <9 s, the signal was of low quality, or the recording did not meet the GS requirements. There was a significant difference between the GS and the AI algorithm results (c^2^= 675.49, *p* < 0.001) and between the results of the AI algorithm and of each general pediatrician (*p* < 0.001). The weighted κ was 0.687 between the GS established by the experienced pediatric pulmonologists and the AI algorithm, indicating substantial agreement. However, the values of the weighted κ between each of the general pediatricians and the GS were significantly different (general pediatricians 0.537–0.308), most of which indicated fair to moderate agreement (Table 2). Kendall's W for interrater agreement among the general pediatricians was 0.39 (*p* < 0.001).

**Table 2:** Cohen's kappa between GS and AI algorithm and general pediatricians.

	**AI**	**General pediatricians**					
		**Listener 1**	**Listener 2**	**Listener 3**	**Listener 4**	**Listener 5**	**Listener 6**
		24 years^*^	19 years^*^	22 years^*^	7 years^*^	7 years^*^	6 years^*^
Cohen's kappa	0.687	0.537	0.429	0.308	0.670	0.439	0.306
(95%CI)	(0.640–0.734)	(0.486–0.588)	(0.380–0.478)	(0.257–0.359)	(0.625–0.715)	(0.390–0.488)	(0.255–0.357)

* *Years of work experience*.

### Accuracy, Sensitivity, Precision, Specificity, and F1-Score

The accuracy of the detection of adventitious breath sounds by the AI algorithm and the general pediatricians with respect to the GS were 77.7% and 59.9% (*p* < 0.001), respectively. Table 3 summarizes the performance of the AI algorithm and the general pediatricians in classifying the recordings. Analysis of the performance of the AI algorithm showed that the sensitivity and specificity in the detection of crackles were 81.3 and 94.1%, respectively, with an F1-score of 80.9%. However, when marked by the general pediatricians, the sensitivity and specificity decreased to 47.8 and 77.1%, respectively, while the F1-score was 42.7%. The sensitivity, specificity and F1-score of the AI algorithm in stratifying wheeze were, respectively, 86.4, 83.0, and 80.9%, which were higher than those of the general pediatricians (82.2, 72.1, and 72.5%).

**Table 3:** Sensitivity, precision, specificity, and F1-score for the AI algorithm and the general pediatricians.

	**Sensitivity %**			**Precision (%)**			**Specificity (%)**			**F1-score (%)**		
	**AI**	**General pediatricians**	***P***	**AI**	**General pediatricians**	***P***	**AI**	**General pediatricians**	***P***	**AI**	**General pediatricians**	***P***
Crackles	81.1	47.8	<0.001	80.6	38.6	<0.001	94.1	77.1	<0.001	80.9	42.7	<0.001
Wheeze	86.4	82.2	>0.05	76.0	64.9	<0.001	83.0	72.1	<0.001	80.9	72.5	<0.001
Mean	83.8	65.0	<0.001	78.3	51.8	<0.001	88.6	74.6	<0.001	80.9	57.6	<0.001

When the collection points on the chest were compared, there was no significant difference in the accuracy of the AI algorithm in the recognition of breath sounds collected from different locations (χ^2^= 1.178, *P* = 0.947), and the overall accuracy was approximately 75% (Table 4). The results of the AI algorithm analysis were compared for the different patient age groups. The accuracy of the AI algorithm was highest among children younger than 12 months; additionally, the F1-score was highest in the recognition of crackles and wheeze in this age group (Table 5).

**Table 4:** Analysis of the performance of the algorithm by chest location.

**Chest location**	**Recordings (*n*)**	**Accuracy (%)**	**Crackles**				**Wheeze**			
			**Sensitivity (%)**	**Precisio*n* (%)**	**Specificity (%)**	**F1-score (%)**	**Sensitivity (%)**	**Precision (%)**	**Specificity (%)**	**F1-score (%)**
**Posterior**										
Upper left	104	74.7	92.9	76.5	93.4	83.9	83.3	62.5	76.5	71.4
Upper right	110	77.8	77.3	81.0	93.2	79.1	85.7	72.7	83.0	78.7
Lower left	100	76.0	88.9	84.2	94.7	86.5	68.4	54.2	80.4	60.5
Lower right	101	80.0	68.8	78.6	94.4	73.3	93.1	75.0	78.0	83.1
**Anterior**										
Upper left	105	78.4	73.7	83.4	94.5	77.8	82.6	73.1	86.3	77.6
Upper right	107	79.8	77.3	85.0	94.7	81.0	85.7	77.4	86.3	81.4

**Table 5:** Analysis of the performance of the algorithm by patient age group.

**Patient age (months)**	**Recordings (*n*)**	**Accuracy (%)**	**Sensitivity (%)**	**Precision (%)**	**Specificity (%)**	**F1-score (%)**
<12	321	81.3				
Crackles			86.5	85.7	93.1	86.1
Wheeze			84.1	84.7	86.5	84.4
12–60	234	74.4				
Crackles			78.0	45.3	93.3	74.4
Wheeze			70.7	45.3	81.9	55.2
>60	72	72.2				
Crackles			50.0	70.0	94.8	58.3
Wheeze			100.0	46.2	76.7	63.2

## Discussion

In this study, breath sound recordings were collected in a real clinical environment, with the typical noises, crying, speaking voices and child movements inherent therein. The AI algorithm was able to fully analyze 93.3% of the recordings, with an accuracy mostly similar to that of the GS established by experienced pediatric pulmonologists. In another verified clinical study of 552 auscultatory sounds from 50 pediatric patients with an average age of 8 years old, the sensitivity, specificity and F1-score of the AI algorithm in distinguishing crackles and wheeze were 83.9 and 78.2%, 79.3 and 57.5%, and 64.6 and 66.4%, respectively. The sensitivity of the AI algorithm was similar to that of the present study, but both the specificity and F1-score were lower than our results. This may be associated with the different feature extraction methods and AI algorithms employed in the two studies (13). Furthermore, our research found that the F1-score decreased with increasing age, so the age differences in the studies may be related to the differences in the evaluation parameters of the models.

There is no doubt that the recognition of adventitious lung sounds is subjective, and the accuracy of the results is highly associated with the specialty and clinical experience of the physician. The lack of an objective standard for evaluating breath sounds restricts the development of relevant clinical research. In the field of heart auscultation, one study implemented the findings of three cardiologists as the GS to quantify the utility of electronic stethoscopes and hand-held echoes in the evaluation of heart murmurs (14). In a study that used breath sounds to validate the diagnostic accuracy of an AI algorithm for interstitial lung disease for rheumatoid arthritis patients, high-resolution CT was used as the gold standard (15). This would have been the best way to produce an objective indicator for use as the standard for the present research; the most closely related examination for lung auscultation is radiological examination, but it is inappropriate to use the results of radiology as the GS for children with common pulmonary disease. The ideal way to establish a gold standard is to have an experienced pediatric pulmonologist at the bedside to immediately analyze and judge the breath sounds collected by the electronic stethoscope. However, the number of experienced pediatric pulmonologists in our hospital is very limited and was unable to meet the needs of our study; therefore, we ultimately chose the current method. In this study, we recruited pediatric respiratory specialists with at least 10 years of work experience to form an expert group. The inclusion criterion for the recordings for entry into the GS database was a consistent evaluation of the result by at least 3 pulmonologists, which is more stringent than previous studies (13). Finally, the GS was used to test the ability of the AI algorithm and general pediatricians to detect adventitious breath sounds. Through the establishment of a GS, we have solved the difficulties in evaluating and comparing different methods or physicians in terms of breath sound detection.

It was found that the recognizability of children's adventitious breath sounds, including crackles and wheeze, of the AI algorithm was higher than that of the general pediatricians. The above results show that even after completing pediatric resident training and achieving more than 5 years of pediatric clinical experience, the pediatricians were unable to match the performance of the AI. Another study consistently found that pulmonologists performed better than other specializations, and interns and pediatricians performed second only to medical students and other specializations. In general, physicians, except for pulmonologists, were no better than medical students (5). A study evaluating the discrimination of breath sound recordings found that the ability to detect stridor was inversely related to work experience (16). Breath sounds, as one the most important physical signs, play an important role in identifying pulmonary disorders in children. It has been found that adventitious sounds, especially crackles and wheeze, have a suggestive effect for many diseases; for example, wheeze can indicate an asthma attack (17), crackles are related to the presence of pneumonia (1), and so on. Therefore, failure to recognize breath sounds correctly will have adverse effects on pediatric clinical work, which may lead to incorrect or delayed diagnosis and treatment of a disease and excessive dependence on radiological examination, including chest X-ray or CT, resulting in the waste of medical resources and other issues.

In terms of medical education and training, a study on training auscultation skills through the use of simulations found that short individual training sessions on a patient simulator significantly improved heart auscultation skills but not lung auscultation skills (18). It is unrealistic to expect that short-term training will improve pediatricians' auscultation skills, so we need to find other, faster, and more direct and convenient ways to help them. A digital stethoscope can collect breath sounds and convert the sound signals into digital signals for saving, sharing, or remote transmission (19). In China, given the very large number of patients, it is impossible to transmit data to specialized hospitals and then have a specialized physician manually discriminate the data one by one; consequently, the needs of community health institutions cannot be met. Therefore, in this study, we use an AI algorithm to train a model to distinguish adventitious sounds and improve diagnostic efficiency. Although the AI algorithm we used is not perfect at present, its performance was at least superior to that of junior residents and general pediatricians with many years of work experience and is consistent with other studies (13). The AI algorithm, as an element of clinical intelligent assistance, can help general pediatricians improve their diagnostic ability and treatment decision making in the future.

The breath sounds were collected in a real clinical environment, and 88.4% of the patients were preschool children in this study. The prevalence of pulmonary diseases in preschool children is relatively high, so it was of great clinical value to verify the AI algorithm for identifying the adventitious breath sounds of children at this stage. Previous studies tended to be limited to school-age children, even adolescents (20), or used standard breath sounds downloaded from a website (21) to evaluate the AI algorithm, which can result in limited research conclusions that cannot be generalized to other pediatric clinics.

By comparing the performance of the AI algorithm at different points on the chest wall, we found no significant differences in the accuracy and F1-score. These results may be useful in developing an optimized clinical panel of breathing sound collection for children. The AI algorithm was designed to perform remote analysis; data were uploaded to the cloud server only by nurses who underwent brief training and took part in this programme. This suggests that the remote analysis can be realized in the clinical process in the future.

This study has some limitations. Among real pediatric respiratory inpatients, the percentage of children over 60 months of age tends to be the lowest; consequently, the fewest number of breath sounds was collected from that group in the study, which may have led to the low accuracy from the algorithm for this age group. The sample population could be expanded, especially to children who are older than 60 months. The pediatricians participating in the study were limited to those practicing in Shanghai and do not reflect the auscultation ability of pediatricians in other regions of China. Therefore, a multi-center study should be carried out in the future, and more experienced pediatric pulmonologists can become involved in the project, at which time the idealized gold standard may be feasibly established. Due to the limitations of our current AI algorithm, we were unable to recognize the crackles and wheezes in the breathing sounds simultaneously, which may have affected the judgment of certain pediatric lung diseases. In future research, we will further improve the AI algorithm to meet the clinical requirements. The study focuses on the accuracy of general breath sound detection; one of our future research directions will combine breath sound detection with a specific pediatric pulmonary disease to build a model for disease diagnosis or follow-up.

In conclusion, it is possible to use an electronic stethoscope to collect breath sounds from children with lung diseases in a real clinical environment and transmit them to specialists for further identification. The accuracy of the AI algorithm in discriminating breath sounds collected at different locations on the chest wall is approximately 75%, which can provide a basis for the design of breath sound acquisition panels for other studies. The ability of the AI algorithm to recognize breath sounds in children is similar to that of a group of experienced pediatric pulmonologists and better than that of general pediatricians from community health service centers, especially in infants younger than 12 months. We will further explore the AI algorithms to recognize crackles and wheezes that occur simultaneously, distinguish between monotonic and polyphonic wheezes, and locate the breathing sounds in the respiratory cycle to ensure that the algorithms are more suitable for real-world clinical application in the future. The combination of an electronic stethoscope with an AI algorithm can potentially be implemented in community health service centers and clinics in the future and may improve the lung auscultation ability of general pediatricians.

## Data Availability Statement

The raw data supporting the conclusions of this article will be made available by the authors, without undue reservation.

## Ethics Statement

The study protocol was approved by the Institutional Review Board of SCMC (approval No. SCMCIRB-K2019056-1). Written informed consent to participate in this study was provided by the participants' legal guardian/next of kin.

## Author Contributions

L-BZ, YY, and JZ contributed to the conception, design of the study and revised the draft critically for important intellectual content. JZ and S-JL contributed to the data analysis. JZ and H-YZ contributed to drafting the submitted article. S-JL contributed to the statistical data analysis. H-SW, BD, LZ, FZ, Y-FW, S-HY, M-YT, W-FD, JL, MC, and XT contributed to the data acquisition and interpretation of the outcomes. L-BZ and YY contributed to crucial revisions of the draft for important intellectual content and providing final confirmation of the revised version to be published. All authors contributed to the data analysis, drafting of the manuscript, and amending of the paper and are responsible for all aspects of the work. All the data could be accessed by all of the authors to assure the accuracy of the reported data. Tuoxiao Intelligent Technology Company provided the electronic stethoscope equipment and AI algorithm analysis at no cost for this study. The researchers and projects have not received any other commercial funding from the company.

## Conflict of Interest

H-YZ was employed by Tuoxiao Intelligent Technology Company. The remaining authors declare that the research was conducted in the absence of any commercial or financial relationships that could be construed as a potential conflict of interest.
